# School nursing: New ways of working with children and young people during the Covid‐19 pandemic: A scoping review

**DOI:** 10.1111/jan.15504

**Published:** 2022-12-21

**Authors:** Georgia Cook, Jane V. Appleton, Sarah Bekaert, Tikki Harrold, Julie Taylor, Dana Sammut

**Affiliations:** ^1^ Centre for Psychological Research Oxford Brookes University Oxford UK; ^2^ Formerly OxINMAHR (Oxford Institute of Nursing, Midwifery and Allied Health Research), Faculty of Health and Life Sciences Oxford Brookes University Oxford UK; ^3^ Oxford School of Nursing and Midwifery, Faculty of Health and Life Sciences Oxford Brookes University Oxford UK; ^4^ Oxford Health NHS Foundation Trust, EOHC Oxford UK; ^5^ School of Nursing and Midwifery Institute of Clinical Sciences, University of Birmingham Birmingham UK; ^6^ Birmingham Women's and Children's Hospitals NHS Trust Birmingham UK

**Keywords:** adolescent health, family care, health services research, literature review, public health nursing, school nursing

## Abstract

**Aim:**

To examine how school nurse practice evolved as a result of the Covid‐19 pandemic.

**Design:**

A scoping review of international literature, conducted and reported in line with Arksey and O'Malley's (2005) framework.

**Data Sources:**

Searches were conducted in September 2021. Ten databases were searched: The British Nursing Database, CINAHL, Cochrane Library, Consumer Health Database, Health and Medicine, Nursing and Allied Health, Public Health, PsycINFO, PubMed and Web of Science. Relevant grey literature was identified through hand searching.

**Review Methods:**

A minimum of three reviewers independently screened articles and two reviewers independently undertook data extraction, with any decisions made collaboratively with the wider team. Much of the literature was not empirical work and so it was not possible to apply a traditional quality appraisal framework.

**Results:**

Searches identified 554 papers (after deduplication) which were screened against title and abstract. Following the full‐text review, 38 articles underwent data extraction and analysis. The review findings highlighted that school nurses adapted their practice to ensure they were able to continue providing their formal and informal school health offer to children, young people and their families and continued working closely with the multidisciplinary team. In addition, the expanded public health role generated by Covid‐19 for school nurses' work was considerable, multi‐layered and added to their routine workload. School nurses displayed resilience, adaptability and creativity in their response to delivering services during Covid‐19.

**Conclusion:**

School nurses took on a leading public health role during the Covid‐19 pandemic. Some developments and practices were highlighted as beneficial to continue beyond the pandemic. However, formal evaluation is needed to identify which practices may merit integration into routine practice. Continued investment in staff and infrastructure will be essential to ensuring school nurses continue to expand their practice and influence as public health experts.

## 1 INTRODUCTION

This review presents a global perspective on the responses of school nurses (SNs) to the public health challenges of the Covid‐19 pandemic. During the pandemic, SNs faced the challenge of delivering remote routine statutory and non‐statutory services and multidisciplinary working, as well as an increased infection control and safeguarding role. This was alongside additional constraints on staffing through sickness and redeployment and personal additional caring and/or home‐schooling responsibilities. Reports show that SNs responded positively and proactively to these demands, and there are many examples of SNs taking on new or expanded public health roles, as well as using creative and innovative practice to overcome barriers. This review brings these examples and evidence together from the literature to give an overview of new, adapted and extended practice, and the benefits and challenges of the changes required. Findings from this review highlight the specialist public health role of the SN. The review strengthens the evidence base regarding SN practice, which can in turn inform policy regarding the vital public health role of the SN, and be a resource for SNs regarding effective practice.

## 2 BACKGROUND

The Covid‐19 pandemic led to remote curriculum delivery for many children across the globe over a significant period of time. This was followed by ongoing disrupted on‐site schooling due to isolation requirements and/or sickness. SNs' informal and formal in‐person contact with children, young people, their families and the multi‐disciplinary team (MDT) was initially halted, and then interrupted with the ensuing changes in social contact guidance. For SNs this has been a significant challenge for maintaining both universal and targeted support for children and young people (CYP) as usual modes of contact through school drop‐in sessions, classroom activities and in‐person professional meetings were no longer viable. As lockdown extended, and isolation and shielding became a regular occurrence, it became apparent that safeguarding CYP was also a significant public health issue as child protection concerns and referrals increased. Several reports highlighted the increased vulnerability of CYP during the mandated lockdown periods (Green, 2020; United Nations [UN] Women, 2020; Young Minds, 2021).

This review presents a synthesis of change in school nursing practice in the international literature as a result of Covid‐19, lockdowns and decreased in‐person contact. The review included all direct practice delivery changes. It also sought to specifically foreground change in practice that relates to SNs' safeguarding work with CYP, their families, and the MDT. A global perspective was taken to facilitate an understanding of how SNs across nations responded to challenges in practice incurred by the pandemic, and widen the learning landscape.

## 3 THE REVIEW

### 3.1 Aim(s)

This review identified the ways in which SN practices evolved as a result of the Covid‐19 pandemic. We documented how SNs worked with CYP and their families, the wider SN community and the MDT. In doing so, we identify the benefits and challenges in SNs' new working practices. Recommendations are made to inform future practice in CYP's public health care; strengthen service delivery in the longer term; and inform policy going forward.

### 3.2 Design

This review was registered with PROSPERO (CRD42021296878). Throughout the design and conduct of the review, expert stakeholders (practising SNs, representatives of professional SN organizations) were consulted. The intention had been to undertake a systematic review to provide a quality appraisal of research evidence; however, due to the low number of empirical studies and wide range of includable grey literature, a scoping review was conducted, in line with Arksey and O'Malley's (2005) framework. As there was limited literature focusing on the impact on vulnerable children, a broad definition of ‘vulnerable’ to encompass all children (in addition to those requiring mandatory support and monitoring) was adopted. This decision was made in consideration of the social, economic and psychological challenges incurred by Covid‐19 which increased the vulnerability of all CYP (Young Minds, 2021). The review is reported in line with the PRISMA guidelines for scoping reviews (Tricco et al., 2018).

### 3.3 Search methods

A broad initial search strategy was adopted due to an awareness that a range of different publication types may constitute relevant literature (i.e. providing examples of new ways SNs worked with CYP as a result of Covid‐19). The framework for our searches was based on population: school nurses and condition/exposure: Covid‐19. A range of specific terms within each category were identified. Search filters included the publication being available in English and published between 2019 and 2021.

Searches were conducted in 10 electronic databases, supplemented by hand searching a range of grey literature associated with SN professional organizations. Where full‐text articles were not accessible, the authors were contacted directly to request access. The full search strategy is provided in Supplementary File S1.

### 3.4 Eligibility criteria

#### 3.4.1 Inclusion

- All types of publications
- Sample/focus is SNs
- An actual change in SN practice
- Change in practice is the result of Covid‐19

#### 3.4.2 Exclusion

- Publication does not clearly include SNs
- Change in practice was not the result of Covid‐19
- Publication does not report a specific change in practice but more generally discusses what could/should have happened

### 3.5 Search outcome

**FIGURE 1:**
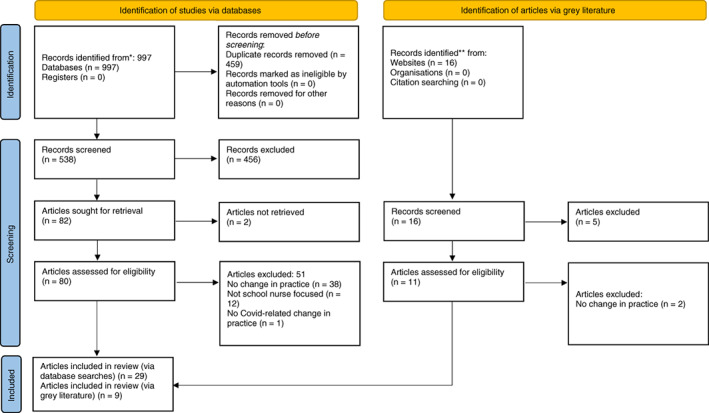
PRISMA 2020 flow diagram showing included searches of databases and grey literature. *Databases: British Nursing Database, CINAHL (EBSCO), Cochrane Library, Consumer Health Database (ProQuest), Health and Medicine (ProQuest), Nursing and Allied Health (ProQuest), Public Health (ProQuest), PsycINFO, PubMed and Web of Science (Clarivate). **Grey literature: School and Public Health Nurses Association (SAPHNA) and Community Practitioner (Community Practitioners' & Health Visitors' Association journal), Public Health England (PHE), Department of Health (DH), National Society for the Prevention of Cruelty to Children (NSPCC) and Early Intervention Foundation. *From*: Page MJ, McKenzie JE, Bossuyt PM, Boutron I, Hoffmann TC, Mulrow CD, et al. The PRISMA 2020 statement: an updated guideline for reporting systematic reviews. BMJ 2021;372:n71. doi: 10.1136/bmj.n71. For more information, visit: http://www.prisma‐statement.org/

One thousand and thirteen references were identified from database searches (*n* = 997) and grey literature searches (*n* = 16). After deduplication five hundred and fifty‐four references were included. After screening by title and abstract, 93 records were assessed as eligible for full‐text review (*n* = 82 database and *n* = 11 grey literature). We excluded those articles where a full‐text version could not be retrieved (*n* = 2), leaving 91 records for full‐text review, following which 38 articles were included (see Figure 1).

### 3.6 Screening

One reviewer (GC) undertook an initial filter of duplicates. A minimum of three of the four reviewers (GC, JA, SB and DS) independently screened all titles and abstracts and then full‐texts of the identified relevant publications. Disagreements were resolved through discussion between the reviewers. During this process, pragmatic decisions had to be made around certain issues that lacked clarity within the articles. These decisions were made collaboratively, with at least three reviewers being involved in all inclusion decisions. The software package Rayyan was used to record screening decisions and justifications (Ouzzani et al., 2016).

We initially sought to capture innovative practice with a specific focus on CYP. However, early engagement with the literature highlighted accounts of diverse adaptations and extensions of existing practice, as well as discussion of the benefits and challenges to SNs fulfilling their role within the constraints of the pandemic. We therefore felt it was important to maximize the opportunity to document the full extent of what SNs were doing in their work with CYP, families and their partnership relationships.

### 3.7 Quality appraisal

Much of the available literature obtained through our searches was not empirical research, therefore it was not possible to apply a traditional quality appraisal framework.

### 3.8 Data abstraction

Following initial familiarization with the literature during a parallel phase of the project (survey development), subheadings were chosen for data extraction based on (1) topics and issues that were prominent in the literature, (2) the content we thought to be most appropriate for meaningfully answering our research questions and (3) input from our steering committee. Once the final list of 38 includable articles was identified, the relevant data from each article were extracted onto an Excel spreadsheet. The data extraction form was piloted using a sub‐sample of 20 articles to ensure it was easy to use, able to be interpreted consistently and captured all relevant information.

The data extraction headings were developed through a preliminary iterative process by the two data extractors (GC and DS) (see Table 1 for extraction headings). Data were extracted from each paper by one of two reviewers (GC or DS) with the other data extractor independently checking the extracted data for rigor and quality. Pragmatic decisions, uncertainties or disagreements were resolved through discussion between the independent reviewers in the first instance and wider project team as necessary.

**TABLE 1:** Data extraction table

Article details		Change in practice						Feedback	
Author(s), publication date	Source; Article type; Location	Work with children and young people	Work with parents	Work with school‐based colleagues	Work with SN colleagues/peers	Partnership working	Innovation	Benefits	Barriers to professional practice
Barbee‐Lee, Seymour, Hett, Norris, Stack, Cartier, Haycox, Armstrong, & Herbert, September 2021	NASN School Nurse; Commentary; Santa Fe, New Mexico, USA	‐ Maintained social distancing, wore masks, and sanitised surfaces while conducting in‐person routine screening (i.e. hearing, vision and dental) ‐ Conducted routine screening consultations via questionnaire with parents ‐ Dental referrals provided for students with concerns, in lieu of oral screenings ‐ Some aspects of sexual health education converted to an online platform ‐ Created and used a virtual nurse's office (accessible to students) ‐ Enforced school exclusions (per state guidelines) ‐ Wore PPE when working with symptomatic students or administering nebulizer treatments ‐ Identified isolation rooms on campus ‐ Drive‐up vaccine clinics held at the district office (routine vaccinations) ‐ Coordinated with the district office to schedule students’ routine vaccination appointments ‐ Liaised with the district's Office of Student Wellness to secure empty rooms at the district office to conduct in‐person student screening appointments	‐ Participated in virtual Q&A sessions to answer questions related to new procedures and student safety ‐ Developed infographics with key information for families (Covid‐19‐related content) ‐ Created and used a virtual nurse's office (accessible to parents) ‐ Conducted hearing, vision and screening consultations via questionnaire with parents	‐ Created and used a virtual nurse's office (accessible to staff)	N/A	N/A	‐ Created the Daily Screening Tool form that school staff used to report self‐screening responses ‐ Supported the development of a HIPAA‐compliant standardised student wellness check‐in form, completed daily by students (and reviewed daily by school wellness teams)	N/A	N/A
Booher, summer 2020	The Alaska Nurse; Commentary; Alaska, USA	‐ Remote communication with students during home working ‐ Taught students ways to stay safe, limit screen time, and make healthy choices ‐ Developed lesson plans to present in classrooms (Covid‐19‐related content, e.g. handwashing)	N/A	‐ Supported custodians with proper disinfecting processes	N/A	‐ Served as a “professional link” between the school district, health department, and emergency operations center	N/A	N/A	N/A
Bullard, McAlister & Chilton, March 2021	NASN School Nurse; Commentary; Texas, USA	N/A	N/A	N/A	N/A	N/A	‐ Developed an electronic pass system to use on campus to control the flow of students requiring nursing assistance	N/A	N/A
Cogan, 2021	Journal of Psychosocial Nursing; Commentary; USA	N/A	N/A	N/A	‐ SN support groups were held twice weekly, via Zoom, to share experiences of being on the frontline of Covid‐19 in school settings	N/A	N/A	N/A	‐ "Bearing the brunt" of parental refusal to comply with indoor masking, negative social responses to Covid‐19 vaccinations, intimidation, bullying regarding quarantine requirements, and lack of cooperation with contact tracing protocols
Combe, July 2020a	NASN School Nurse; Commentary; USA	‐ Checked in on students with known health conditions (via telephone) ‐ Outdoor distribution of instructional materials (no further details provided) ‐ Outdoor medication pick‐up points ‐ Dropped by teachers’ remote classrooms to connect with students ‐ Produced a health promotion video addressing student fears about nurses and other health providers in PPE ‐ Used social media to communicate with students to let them know they were missed and to encourage them to practise self‐care	N/A	‐ Collaborated with school counsellors to produce a resource list to meet families’ needs ‐ Worked with teachers to identify students at risk for chronic absenteeism	‐ State SN organization held virtual town halls to connect with members (at times on a weekly basis)	‐ Worked with school food service partners to ensure that students dependent on school nutrition continued to have their needs met through drop offs and deliveries	N/A	‐ Time to research unique student health conditions, collaborate with paediatricians and other healthcare providers, and update Individualised Healthcare Plans without interruptions [relating to time spent working from home] ‐ SN garnered a “seat at the Executive Team table”	‐ Managing work and family responsibilities within the same space and time, e.g. juggling multiple curbside grocery pick‐up services, trying to stock up on essentials
Combe, November 2020b	NASN School Nurse; Commentary; USA	‐ Covid‐19 monitoring, tracing and quarantining	‐ Notified parents about Covid‐19 cases (via telephone) ‐ Sent weekly reminder emails asking parents to screen their child(ren) daily	‐ Identified positive cases among school staff ‐ Enacted quarantines or school closures as appropriate ‐ Supported education staff to devise plans to keep classrooms safe	‐ Provided professional peer support in the SchoolNurseNet community and on social media	N/A	N/A	N/A	‐ Covid‐19‐specific responsibilities impacted on time to do 'traditional' SN role ‐ Navigating different informational sources ‐ Ever‐changing standards of care, limited resources, the unknowns about Covid‐19 transmission ‐ Unfamiliar work environments for those new to school nursing ‐ Balancing professional practice and judgement with wider guidance ‐ Staffing levels ‐ Workload expectations and increased working hours ‐ The need to travel between schools ‐ “Everyone's need for information at a moment's notice 7 days a week" ‐ Limited PPE supply chain access for schools ‐ Fear of contracting and spreading Covid‐19 ‐ Stakeholders “forgot” about ongoing non‐Covid‐19 needs of many students
Driscoll, Hutchinson, Lorek & Kiss, June 2021	SAPHNA; Empirical research (modified Delphi study in two stages: (1) interviews with 67 safeguarding lead professionals in London, and (2) national survey of the same professional groups (417 survey responses across England). NB. SNs were not involved as participants but were referred to in the report by other participating professionals); England, UK	‐ Arranged to see young people in parks and outdoor areas	N/A	N/A	N/A	N/A	N/A	‐ Seeing CYP in parks and outdoor areas improved attendance at appointments; one nurse noted that “many of these were children who wouldn't have turned up to appointments in school time pre‐lockdown"	N/A
Evans, November 2020	Nursing Children and Young People; Commentary; UK	‐ Used other venues such as children's centers ‐ Walk‐and‐talks ‐ Change in organization of appointments; greater reliance on bookings than drop‐in services ‐ Vaccination backlogs; marquees set up in school fields and mobile units (e.g. use of the local football stadium and drive‐through clinics) ‐ ChatHealth introduced in April [2020]; most used in times of lockdown, less use as restrictions eased	‐ Moved to online meetings ‐ Launch of ChatHealth in April [2020], used by parents of primary school children	‐ Moved to online meetings with teachers	N/A	N/A	N/A	‐ Feedback on walk and talks was positive: "Being outside in the fresh air, among nature, is very calming” (feedback from CYP and families, as reported by a SN); “It brought a new dimension to the support we provide. It is a valuable environment to use to engage in a calm manner that we previously may have overlooked, as we traditionally meet pupils indoors” (SN)	‐ SNs required to wear full PPE while in close proximity to children, and some schools required SNs to wear full PPE "all the time" ‐ PPE created a "barrier to building relationships with pupils". ‐ Lack of ability to see facial expressions and inability to touch arm to show empathy cited as barriers ‐ Workload increase
Fauteux, August 2021	American Journal of Nursing; Commentary; USA	‐ When schools closed, SNs resumed carrying out many regular duties remotely: providing basic health education online, connecting families to services for their health and social needs, and doing what they could to help students with chronic conditions stay healthy at home ‐ Outdoor provision of advice and information to students ‐ Outdoor distribution of food and school supplies ‐ Visited skate parks with water, snacks and helmets to connect skaters with mental health support, tutoring, and other resources	N/A	N/A	‐ Twice weekly support groups held for SN peers	‐ Assisted public health departments to investigate disease outbreaks in the school community and beyond ‐ SN team used a refurbished vehicle to conduct Covid‐19 testing and help at Covid‐19 vaccination clinics	‐ Collaborated with computer services team to modify the district's document sharing platform to reduce the administrative burden of contact tracing	‐ Covid‐19 collaboration strengthened the relationship between SNs and public health officials	‐ Increased workloads 'tremendous' (reported 12‐14‐hour days, seven days a week); "they're exhausted"
Ferrara, February 2021	Massachusetts Report on Nursing; Commentary; Massachusetts, USA	‐ Change in practical physical setup: medical waiting room (where students with Covid‐19 symptoms isolated) and triage area (outside SN office for assessing students' symptoms prior to determining whether they could enter the office) ‐ CYP required masks, social distancing and hand sanitiser to enter SN office	N/A	N/A	N/A	N/A	N/A	N/A	N/A
Flaherty, November 2020	NASN School Nurse; Commentary; Massachusetts, USA	‐ Taught health lessons virtually	‐ Met virtually with parents	‐ Virtual meetings with peers, interdisciplinary staff, and administration (including Section 504 and Individualised Education Plan meetings)	N/A	N/A	N/A	‐ Partnering with Local Boards of Health around Covid‐19 elevated the critical role and profile of SNs	N/A
Gingell & Spencer (no date)	SAPHNA; Service review; Cambridgeshire, England, UK	‐ Telephone consultations used initially, replacing face‐to‐face for “non‐essential services” ‐ Video interactions used for face‐to‐face digital interventions	‐ Telephone consultations used initially for families, replacing face‐to‐face for “non‐essential services” ‐ Video interactions used for face‐to‐face digital interventions	N/A	N/A	‐ Microsoft Teams made available ‐ Norfolk Healthy Child Programme used video conferencing for daily sitrep, huddle and wider multi‐agency meetings	N/A	‐ Video conferencing described as beneficial for flexible working; saved time and travel costs; and resulted in increased attendee numbers at meetings ‐ CYP and families reportedly “engaged positively” during video consultations ‐ Virtual visits allowed child welfare checks without needing doorstep visits to shielding families	‐ Initially contacts were conducted via telephone but these were deemed "insufficient for their completeness"
Hansen, May 2021	NASN School Nurse; Commentary; Lee's Summit, Missouri, USA	N/A	N/A	‐ Daily interactions between SNs and building administrators ‐ SNs worked closely with school principals in the collection and utilisation of data	N/A	N/A	‐ Developed a Covid‐19‐specific hotline (and data sharing process) for schools to report data to the district	‐ Daily interactions strengthened the relationship between the school nurses and the building administrators ‐ Increased interaction with building administrators increased the visibility and value of SNs and SN data ‐ Highlighted role of schools in larger public health team and the critical role SNs play in the team	N/A
Hoke, Keller, Calo, Sekhar, Lehman, & Kraschnewski, February 2021	The Journal of School Nursing; Empirical research (cross‐sectional internet survey of Pennsylvania, *n* = 350 SNs); Pennsylvania, USA	N/A	‐ In a cross‐sectional internet survey, SNs (*n* = 350) reported that they: ‐ Switched to only electronic communication (in place of paper communication) = 144 or 41.1% ‐ Increased communicate with parents to check in on students’ health = 147 or 42.0% ‐ Reduced all forms of parent communication = 71 or 20.3% ‐ Delayed communication regarding school immunisations = 128 or 36.6% ‐ Increased communication regarding Covid‐19 = 173 or 49.4%	N/A	N/A	N/A	N/A	N/A	N/A
Kunz, Manno, Ruffatti, Blohm, Wuerger, Keegan, & Karras, March 2021	NASN School Nurse; Commentary; Illinois, USA	N/A	N/A	N/A	N/A	‐ Weekly conferences between local department of health and school staff (including SNs) to share Covid‐19 guidelines, address questions and present county data ‐ Created School Nurse Task Force (SNTF), which met weekly on Zoom to provide county‐wide implementation of Covid‐19 protocols for school health offices and classrooms ‐ SNTF created and shared a “COVID‐19 toolkit" (20‐plus page toolkit containing information on Covid‐19 for parents, students and staff) ‐ Created protocols and documentation for contact tracing, isolation and quarantine in the school setting	‐ Developed worksheets for symptomatic staff/students and close contacts using checklist format ‐ Developed online contact tracing forms to facilitate reporting ‐ Created a visual calendar for patient instruction to track return dates ‐ Developed a release verification letter to ensure requirements were met before returning to the classroom or work ‐ Created post‐vaccine symptom management guidance	N/A	N/A
Lee, West, Tang, Cheng, Chong, Chien, & Chan, June 2021	Nursing Outlook; Empirical research (qualitative study using semi‐structured interviews, *n* = 19 SNs working in Hong Kong); Hong Kong	‐ Communicated with students via telephone ‐ Monitored students’ compliance with guidelines ‐ Created an isolation area (no further details provided) ‐ Reinforced the need for 14‐day quarantine periods for returned travellers, checked temperatures upon arrival, and monitored student hygiene	‐ Communicated with parents via telephone ‐ Increased contact to support parents of children from “special school” ‐ Educated parents on the need to check students’ temperatures before sending them to school and to keep them home if unwell	‐ Provided psychological support to school staff ‐ Contacted staff members to provide health advice ‐ Provided staff with education in areas such as proper hand hygiene, cleaning and disinfecting of the school and school buses, wearing of masks, proper cleaning of vomitus, and disposal of PPE	‐ Formed a WhatsApp group to facilitate informal communication with other SNs, to help overcome professional isolation, information deficit, and uncertainty	N/A	‐ Developed a triage flowchart	‐ Telephone contacts were reportedly well received by students ‐ Technology enabled SNs to stay up‐to‐date with information and allowed ongoing communication with professionals ‐ Increased visibility, appreciation and respect for SNs’ professional image led to improved relationships with school staff and parents, and improved ability to deliver health education role ‐ SNs “positioned advantageously against budget constraints and possible job cuts” ‐ SNs who reported feeling respected were reportedly more satisfied with their jobs, more grateful for their employers, more resilient, cooperated more with others, performed better, and were more likely to take direction from leaders	‐ Lack of information and guidelines, and confusing messages from the government caused SNs stress ‐ SNs reportedly received fewer resources and updates than hospital settings ‐ Challenges of obtaining and maintaining appropriate PPE stocks (and knock‐on impact of this on stress and uncertainty for SNs) ‐ Challenge of social distancing in small schools ‐ Challenge of maintaining hygiene practices among younger student cohorts as well as issues maintaining PPE practices with students with intellectual disabilities ‐ School teachers refused to wear masks at the beginning of the pandemic, and “bargained” with SNs to relax the rules
Marrapese, Gormley, & Ceschene, July 2021	NASN School Nurse; Commentary; Greenfield, Massachusetts, USA	‐ Maintained continuity of care by scheduling telehealth assessments in place of scheduled in‐person screening visits ‐ Health education for the student (and family) became the primary prevention tool used in telehealth assessments ‐ Created pre‐recorded informational videos (holistic home health and illness prevention)	‐ Provided health education to families via telehealth assessments and pre‐recorded informational videos	‐ Collaborated with school adjustment counsellors to facilitate social groups, to improve “student connectedness” ‐ Provided monthly education to staff on the importance of sharing electronic student information confidentially	N/A	‐ Collaborated with the Massachusetts Department of Public Health to facilitate contact tracing	‐ Partnered with the IT department to develop password‐secured enrollment portals, electronic consent, and an electronic health record that was independent of the student information system	N/A	N/A
Martinsson, Garmy, & Einberg, June 2021	International Journal of Environmental Research and Public Health; Empirical research (qualitative study using semi‐structured online focus groups and one interview, *n* = 17 SNs working in Sweden); Sweden	‐ Held health dialogues via telephone and video calls ‐ Walk‐and‐talks ‐ Recorded informational videos about health (no detail provided about content)	‐ Increased health dialogues with parents when offering digital call‐in options to participate remotely	‐ Switched to digital meetings for professionals working in school ‐ Cooperation increased within the school health service at upper secondary schools during the period of distance learning	N/A	N/A	N/A	‐ Use of virtual methods for consultations cited as “helpful” (with caveat of need to see the student/use video) ‐ SNs reported being able to gain greater insight/understanding into their students' home situations ‐ Easier to reach guardians as many were working from home ‐ More conversations about serious topics when talking to the students ‐ Digital/remote options resulted in time gain ‐ Positive feedback reported on digital school health service meetings ‐ Some SNs reported receiving good support from their managers during the pandemic	‐ Some SNs felt digital health dialogues make it difficult to transition to discuss sensitive topics ‐ Difficulty of working with students who felt unwell and sought contact via virtual chat platforms ‐ Need to check student was alone when communicating virtually "so that sensitive topics could be dealt with" ‐ Some SNs cited difficulty communicating with guardians with different linguistic background when conveying public health recommendations ‐ Some SNs felt information shared digitally was not as detailed ‐ Closer contact with students resulted in increased demands on SNs’ time ‐ Some SNs felt they lacked support from managers and colleagues ‐ Inconsistent practices across different schools and management ‐ Ethical challenges, such as saying no to children who wanted a hug
Maughan, Johnson, Gryfinski, Lamparelli, Chatham, & Lopez‐Carrasco, January 2021	NASN School Nurse; Commentary; USA	N/A	N/A	‐ SNs reported spending much of their summer “learning, preparing, and communicating” with their education leaders to help make decisions before the new school term	‐ State SN consultants developed materials to help guide SNs, direct them toward latest evidence, and answer questions ‐ Several consultants worked together to draft guidance for healthcare professionals in schools related to PPE, which was reviewed, developed, and finalised with representatives from the NASN	‐ Weekly calls with state experts who guided SNs through various scenarios ‐ In states with a state SN consultant, this individual often organized calls to share evidence, resources and data ‐ Through networking, some SNs spoke at town halls and at school board meetings	‐ A state SN consultant collaborated to develop a system to identify and deliver appropriate PPE to all the schools in North Carolina	‐ SNs’ role and communication with educators helped educators to stay informed and increased the SNs’ credibility	‐ Many SNs had to adjust plans and constantly communicate with educators who still asked for decisions to be made that may not have been based on the best evidence
Park, Cartmill, Johnson‐Gordon, Landes, Malik, Sinnott, Wallace, & Wallin, May 2021	NASN School Nurse; Commentary; USA	‐ Developed school‐located vaccination events (SLVE) for students (and staff) ‐ Produced relevant supporting materials to the development and running of an SLVE, including a template for events and a checklist for future events	N/A	‐ Developed a SLVE for staff (and students)	‐ A group of seven SNs assembled and met weekly via Zoom to identify, evaluate and apply research evidence relevant to the SVLE	‐ Held a virtual meeting with an official from the local department of public health to discuss and implement plans for vaccine clinics in schools ‐ Worked on a general district vaccination plan in consultation with the relevant department of health services ‐ Consulted with school district liability insurer and district attorney prior to finalising plans ‐ A draft plan was forwarded to the health services director; upon amendment it was sent to the superintendent's advisory team for review and approval	N/A	N/A	N/A
Robarge, April 2021	The Bulletin (official publication of the Indiana State Nurses' Association); Commentary; Indiana, USA	‐ Created videos for students (and families) about public health measures ‐ Social media platforms like TikTok were used for teaching hand washing, mask wearing, social distancing and return to school protocols ‐ Participated in food distribution and other assistance for families with social and health disparities	‐ Created videos for (students and) families about public health measures	‐ Involved in the planning, implementing, and evaluating of schools' pandemic and reopening plans	‐ NASN hosted an annual conference in a virtual environment	‐ Many tasked with reporting case numbers to state departments of health ‐ Assisted local health departments with contact tracing, including case investigation of positive cases of students and staff while continuing to provide care coordination	N/A	N/A	N/A
Robinson, February 2021	DNA Reporter (Delaware Nurses' Association); Commentary; Delaware, USA	‐ Conducted daily symptom screenings ‐ Reconstructed office spaces to provide for isolation areas ‐ Performed contact tracing in conjunction with the department of public health ‐ Determined the possibility of quarantine for every report of illness ‐ Relied on ambiguous symptoms to make serious clinical judgements ‐ Educated “everyone” about Covid‐19 ‐ Provided Chromebooks and ensured internet access for students ‐ Virtual provision of mental health counselling sessions and telemedicine	N/A	N/A	N/A	‐ Assisted the department of public health in contact tracing and educating communities	N/A	N/A	‐ Ensuring that families were compliant with medical guidelines was cited as a challenge ‐ SNs took on multiple roles within illness management, with minimal or inadequate PPE, and “little to no additional training”
Schwind, 2020	Texas Nursing Magazine; Commentary; Texas, USA	N/A	N/A	N/A	N/A	‐ Helped district administrators to develop guidance and plans around school reopening ‐ Helped district administrators to understand new terminology (e.g. PPE, mask fit testing, contact tracing)	N/A	N/A	N/A
Sorg, August 2020	The Conversation (independent source of news analysis and comment); Commentary; USA	‐ Undertook daily symptom screening ‐ Assessed illnesses and isolating children as required ‐ Helped to decide whether or not a child should remain at school	N/A	N/A	N/A	N/A	N/A	N/A	‐ New Covid‐19 specific responsibilities added to SNs’ “already heavy workloads" ‐ SNs faced “tremendous stress” as they navigated students’ return to school ‐ Some SNs felt frustrated with the lack of resources and with school plans that they felt put students and staff at risk ‐ Worked out of small offices with little room for social distancing
Tomkinson (no date)	SAPHNA; Case study; Rugby, England, UK	‐ Swapped to a telephone consultation	‐ Used email and post to transfer materials to and from parents/guardians	N/A	N/A	N/A	N/A	‐ Child preferred telephone conduct of the health assessment rather than face‐to‐face ‐ Child engaged well and felt listened to in an environment with limited distractions ‐ SN felt that the child “spoke a lot more than they would have done if the [assessment] had been carried out face‐to‐face within either a clinical setting or school" ‐ SN felt telephone was appropriate for conducting the assessment ‐ SN was able to “hear the positive interaction between the young person and the foster carer” ‐ SN felt able to develop trust and rapport with the child	N/A
Traut, December 2020	The Nursing Voice (official publication of the Illinois Nurses Foundation); Commentary; Illinois, USA	N/A	‐ Educated communities on the proper use of PPE	‐ Educated school staff on the proper use of PPE	N/A	‐ Participated in school district workgroups to create reopening plans, protocols, and procedures ‐ Worked with local health depts for case monitoring and contact tracing ‐ Provided resources and assistance to administrators regarding Covid‐19 cases, school closures and department of health recommendations ‐ Worked with administrators in “creative ways” to source supplies (e.g. PPE) ‐ SNs were “on‐call” for phone conversations with administrators regarding ever‐changing guidelines from the department of public health and the Illinois State Board of Education	‐ Reviewed mass temperature scanners ‐ Developed phone apps for tracking and reporting symptoms in students and staff	N/A	‐ Some SNs felt they were “left out of planning conversations”
Unknown, 2020a	New Hampshire Nursing Association News; Commentary; New Hampshire, USA	‐ Monitored for Covid‐19 symptoms and undertook screening, taking into account child's clinical history ‐ More cautious approach when making clinical decisions	‐ Monitoring children during Covid‐19 required increased communications with parents (via telephone)	N/A	N/A	‐ Received support from state health experts, including weekly video calls ‐ Took part in a virtual town hall meeting with the New Hampshire Chapter of the American Academy of Pediatrics	N/A	N/A	N/A
Unknown, October 2020b	DNA Reporter (Delaware Nurses' Association); Commentary; Delaware, USA	‐ Planned, delivered and presented health education for online learning (no further details provided) ‐ Monitored student symptoms	N/A	‐ Attended online Individual Education Planning meetings for students in special education	N/A	‐ Liaised with administrators and collaborated with staff members to pre‐empt Covid‐19 outbreaks ‐ Had frequent conversations with Division of Public Health to review up‐to‐date guidance from the CDC and WHO ‐ Worked with administrators to plan for staff screening and return to school	N/A	N/A	N/A
Unknown, April 2020c	SAPHNA; Case study; Caldecote, England, UK	‐ Telephone consultations replaced face‐to‐face Early Help meeting ‐ Recommended ChatHealth to a child ‐ Sent a list of useful websites to child in the post	‐ Telephone consultation replaced face‐to‐face Early Help meeting	N/A	N/A	N/A	N/A	‐ SN felt they were able to provide the “same level of care and service” to the child and mother	N/A
Unknown, 2020d	SAPHNA; Case study; Nuneaton, England, UK	‐ Used telephone to conduct annual ‘looked after child’ health review	‐ Used telephone call to conduct annual ‘looked after child’ health review ‐ Used post to send out pre‐appointment questionnaire and appointment details	N/A	N/A	N/A	N/A	‐ SN reported that conducting health assessments over the telephone is “not ideal” but given circumstances, it was the best option to allow the child to share new concerns	N/A
Unknown (no date)	SAPHNA; Case study; Walsall, England, UK	‐ Engaged with child via virtual technology	‐ Provided parents with advice and support via virtual technology	N/A	N/A	‐ Used Microsoft Teams for collaboration with social work managers to review the needs of CYP, and consider how interventions of child protection and child in need plans could be delivered ‐ Conducted virtual case conference, core groups and child in need meetings	N/A	‐ SN felt that virtual technology capability added a dimension to building relationships, opened new channels of communication, and allowed more in‐depth discussions with a focus on the needs of children than before	‐ Covid‐19 resulted in a reduction in services from a specialist provider, resulting in the withdrawal of support for a child and his family
Various, June 2020a	Community Practitioner; Commentary; UK	‐ Used ChatHealth and telephone contacts	N/A	N/A	N/A	N/A	N/A	N/A	N/A
Various, November 2020b	SAPHNA; Collection of case studies; England, UK	‐ Used ChatHealth, telephone and video call consultations ‐ Walk‐and‐talk sessions ‐ Produced a pre‐recorded video about returning to school safely (for students and families)	‐ Care plans were reviewed virtually ‐ Produced a pre‐recorded video ‐ "to spread the message that A&E was safe to visit” (shared on YouTube) ‐ Produced a pre‐recorded video about returning to school safely (for students and families) ‐ Telephone calls ‐ Facilitated rollout of a telephone response service that parents could call for advice and support	‐ SNs involvement in the telephone response service relieved pressure on head teachers who received a high volume of calls ‐ Had regular meetings with head teachers to provide support and advice, and answer questions around Covid‐19	‐ Care plans were reviewed virtually	N/A	N/A	‐ Parents reported positive feedback on walk‐and‐talks ‐ Walk‐and‐talks reportedly filled the gap of missing “physical connection" and allowed SNs to engage with CYP who otherwise struggled ‐ The face‐to face contact provided by walk‐and‐talks s allowed for deeper assessments and more personal meetings ‐ Parents reported positive feedback on SNs’ A&E video, calling it “helpful”, “fun to watch” and “reassuring”	N/A
Waters, March 2021	Community Practitioner; Commentary; UK	‐ Online consultations	‐ Online consultations	N/A	N/A	N/A	N/A	N/A	N/A
White, July 2020a	British Journal of Child Health; Commentary; UK	‐ Used technology and virtual assessments to support children ‐ Where necessary and after risk assessment, provided face‐to‐face, walk‐and‐talk, doorstep and drive‐through services	‐ Used technology and virtual assessments to support families	‐ Used technology to support schools	N/A	‐ Used technology to support “partners”	N/A	N/A	N/A
White, July 2020b	Nursinginpractice.com; Commentary; England, UK	‐ e‐Clinics and brief interventions were held via platforms including Microsoft Teams, FaceTime and WhatsApp ‐ Provided health education/promotion lessons, videos and resources covering topics such as hand/respiratory hygiene to puberty, transition to high school and food poverty	N/A	‐ Provided digital training for school staff regarding medical conditions in schools, such as asthma, and diabetes ‐ Held safeguarding meetings via a range of digital platforms	N/A	N/A	N/A	‐ Many CYP reported preferring virtual contact over face‐to‐face, especially when it offered flexibility in terms of appointment times ‐ Many ‘looked after’ children reported feeling more able to discuss personal and sensitive matters virtually ‐ Virtual contacts enabled SNs to gain a holistic view of the child and family health needs ‐ Young carers appreciated the time and support they were given virtually	‐ Virtual contacts with children and families reportedly took up much more of SNs’ time
White, March 2021	British Journal of Child Health; Commentary; UK	‐ Extended childhood vaccine access during evening and weekend hours, via “catch‐up” sessions, “drive‐thru” sites and at “each and every contact" ‐ One SN delivered immunisations in risk‐assessed gardens	N/A	N/A	N/A	N/A	‐ Introduced and expanded electronic systems, including “e‐consent”, vaccine notifications, and text support services	‐ Introduction and expansion of various e‐systems made services "faster, smoother and more efficient" ‐ Access to e‐systems, such as the Child Protection Information System, helped SNs to “target and thus protect many of the most vulnerable"	‐ Digital poverty was a barrier for CYP and families accessing virtual services
Yip, Yip & Tsui October, 2020	Advances in Nursing and Midwifery; Empirical research (qualitative study using phenomenological design, *n* = 9 SNs working in special schools in Hong Kong); Hong Kong	‐ Prepared online video recordings of the practical demonstration of healthcare techniques, such as hand sanitisation and mask wearing (for students)	‐ Increased contact with parents	‐ Communicated with colleagues using Google Meet and Zoom	‐ Peer support groups formed, mutual mental health support offered	N/A	N/A	‐ SNs became closer with their school colleagues a result of using online platforms such as Google Meet	‐ Infection prevention and control measures “imposed an added burden” on SNs ‐ Hiring of new SNs increased current SNs' workload due to having to teach the role ‐ Increased contact with parents added to workload ‐ Emotional challenges: SNs reported "feelings of helplessness, powerlessness, stress, and frustration when describing how they had felt in this neglected specialty" ‐ Lack of training to undertake expanding role (in relation to infection control duties)

Abbreviations: A&E, Accident & Emergency; CDC, Centers for Disease Control and Prevention; CYP, children and young people; HIPAA, The Health Insurance Portability and Accountability Act; NASN, National Association of School Nurses; PPE, personal protective equipment; SAPHNA, School and Public Health Nurses Association; SN(s), school nurse(s); SNTF, school nurse taskforce; SVLE, school‐located vaccination event; WHO, World Health Organization; NB. Terminology included in the table reflects the terminology used in the original articles.

### 3.9 Synthesis

The headings of our data extraction sheet formed the framework for our synthesis. Two members of the team (GC and DS) independently grouped the extracted data to produce initial themes in the context of each heading (i.e. CYP, parents, etc.). Preliminary groupings were reviewed by another independent reviewer (SB) and extensively discussed (if necessary also with the wider team) until consensus was reached on the final themes.

## 4 RESULTS

### 4.1 Literature characteristics

The included publications were from the United States of America (USA) (*n* = 22), the United Kingdom (UK) (*n* = 13), Hong Kong (*n* = 2) and Sweden (*n* = 1). A range of publication types were represented: commentary (news, opinion pieces, editorials, etc.) (*n* = 27), case studies (*n* = 5), empirical research (*n* = 5) and a service review (*n* = 1). The included articles were published in 2022 (*n* = 1, originally published online in 2021), 2021 (*n* = 17), 2020 (*n* = 15) and some had no date (*n* = 5). See Table 1 for a summary of extracted data.

### 4.2 Thematic findings

As proposed by Bradbury‐Jones et al. (2021), a patterning table, summarizing the key ways SN practice changed and evolved during Covid‐19, is presented in Table 2. The identified patterns, advances, gaps, evidence and research recommendations are presented in Table 3. Themes are organized based upon whether they relate to the ongoing school health offer or the new expanded SN role arising from Covid‐19.

**TABLE 2:** Patterning table showing the ways in which school nursing practice changed and evolved during Covid‐19

	The continued school health offer			The expanded school health offer		
Author and publication year	Engagement ‐ CYP & families	Education	Liaison with professionals	Infection prevention and control ‐ CYP & families	Infection control ‐ broader role	Innovation
Barbee Lee et al., 2021	x Virtual nurse's office; drive‐up clinics; screening questionnaires; empty offices for in‐person screening; establishing isolation areas	x Online lessons; virtual Q&A sessions with parents	x Virtual nurse's office	x Social distancing, masks and sanitisation for in‐person screening; enforcing school exclusions; infographics		x Development of a standardized student wellness form; development of a staff screening tool
Booher, 2020	x Remote communication			x Lesson plans (Covid‐19 and associated issues)	x Supporting custodians with disinfecting processes; serving as a ‘professional link’ between districts and local health centers	
Bullard et al., 2021						x Development of an electronic pass system
Cogan, 2021			x SN support groups (via Zoom)			
Combe, 2020a	x Social media; outdoor distribution of resources; telephone		x Virtual town halls; working with teaching staff to identify students at risk of chronic absenteeism; working with school counsellors to produce resource lists; working with food service partners	x Pre‐recorded informational videos		
Combe, 2020b			x Professional peer support (SchoolNurseNet and social media)	x Covid‐19 monitoring, tracing and quarantining; telephone calls and emails	x Advising staff on infection prevention and control; monitoring social distancing and Covid‐19 cases; actioning quarantines or school closures	
Driscoll et al., 2021	x Meeting CYP in parks and outdoor areas					
Evans, 2020	x ChatHealth; walk‐and‐talks; vaccinating in marquees, mobile units and alternative venues; drive‐through vaccination clinics; online meetings		x Online meetings			
Fauteux, 2021	x Outdoor distribution of resources, advice and information	x Virtual health education	x SN support groups		x Covid‐19 testing, investigation and vaccinations	x Making adaptations to document sharing
Ferrara, 2021				x Change to physical setup; assessing symptoms; masks, social distancing and hand sanitiser required to enter office		
Flaherty, 2020	x Virtual meetings	x Virtual health education	x Virtual meetings			
Gingell and Spencer, n.d.	x Telephone; video interactions for face‐to‐face digital interventions		x Daily sitrep, huddle and wider multi‐agency meetings (via Microsoft Teams)			
Hansen, 2021					x Supporting Covid‐19 data collection and use	x Covid‐19 data sharing and reporting via hotlines
Hoke et al., 2021	x Switch to only electronic communication			x Communication increased due to Covid‐19‐related issues		
Kunz et al., 2022					x Vaccination planning; information sharing; resource development and sharing	x Development of screening, monitoring and quarantine release tools (staff and students); creation of ‘Covid‐19 toolkit’
Lee et al., 2021	x Telephone		x WhatsApp for informal peer communication; providing emotional support to school staff	x Preparing procedures; monitoring student compliance with hygiene practices; maintaining PPE stock; creating isolation areas; monitoring quarantine compliance; temperature checks; increased contact to support parents; telephone calls	x Advising staff	x Development of a triage flow‐chart
Marrapese et al., 2021	x Telehealth assessments	x Pre‐recorded informational videos	x Working with counsellors to improve student ‘connectedness’; educating staff		x Contact tracing	x Development of IT systems (enrollment and e‐consent systems, electronic health record)
Martinsson et al., 2021	x Walk‐and‐talks; telephone; video calls	x Pre‐recorded informational videos	x Digital meetings			
Maughan et al., 2021					x Resource sharing with state experts; collaboration with national SN organization to develop materials; speaking at town hall events; planning for school reopening	x Development of a system to identify and deliver PPE
Park et al., 2021					x Collaboration for school‐based vaccination clinics; general district vaccination planning; virtual peer meetings (via Zoom)	
Robarge, 2021	x Food distribution		x Virtual conference	x Pre‐recorded informational videos; social media (via TikTok)	x Case monitoring and contact tracing; planning for school reopening	
Robinson, 2021	x Providing students with Chromebooks and ensuring internet access			x Daily Covid‐19 screening; establishing isolation areas; contact tracing; managing individual cases; educating on Covid‐19	x Case monitoring and contact tracing	
Schwind, 2020					x Assisting district administrators to plan and develop guidance for students' return to school	
Sorg, 2020				x Daily Covid‐19 screening; enacting isolations; deciding if students should be in school		
Tomkinson, n.d.	x Email; telephone; post					
Traut, 2020				x Education on PPE	x Being ‘on‐call’ for Covid‐19 discussions; education on PPE; case monitoring and contact tracing; acquiring PPE and planning for Covid‐19 scenarios; planning for school reopening	x Development of phone apps for tracking and reporting of student symptoms
Unknown, 2020a				x Covid‐19 monitoring and screening; increased communication (phone calls and conversations)	x Video calls; virtual town hall	
Unknown, 2020b		x Virtual health education	x Online Individual Education Planning meetings	x Student symptom monitoring	x Liaising with administrators and school staff to pre‐empt Covid‐19 outbreaks; liaising with local authorities to review national and international guidance; planning for school reopening	
Unknown, 2020c	x ChatHealth; post; telephone					
Unknown, 2020d	x Telephone; post					
Unknown, n.d.	x Virtual technology		x Virtual (safeguarding meetings); Microsoft Teams			
Various, 2020a	x ChatHealth; telephone					
Various, 2020b	x ChatHealth; video calls; walk‐and‐talks; telephone		x Virtual (reviewing care plans)	x Telephone response service; pre‐recorded informational videos (via YouTube)	x Advising staff	
Waters, 2021	x Online consultations					
White, 2020a	x Walk‐and‐talks; doorstep and drive‐through services; virtual assessments; use of technology generally to support families		x Technology (support to schools)			
White, 2020b	x e‐Clinics; consultations/contact via Microsoft Teams, FaceTime and WhatsApp	x Pre‐recorded informational videos	x Digital platforms (training and meetings)	x Pre‐recorded informational videos		
White, 2021	x Drive‐through vaccine clinics; outdoor contacts; extended evening/weekend ‘catch‐up’ sessions					x Implementing vaccine notifications, online text support services and e‐consent
Yip et al., October, 2020			x Communications via Zoom and Google Meet; peer mental health support	x Increased contact with parents; pre‐recorded informational videos		

Abbreviations: CYP, children and young people; PPE, personal protective equipment; SN, school nurse.

NB. Level of detail provided within this table may vary, reflecting the degree of information provided by the articles.

**TABLE 3:** PAGER table

Patterns	Advances	Gaps	Evidence for practice	Research recommendations
*The continued school health offer*	There is evidence (anecdotal, informal and empirical) of innovative practices developed and adopted by SNs to deliver routine school nursing services.	There is a paucity of empirical research exploring this topic. There is a lack of geographic representation across the literature.	While there is some evidence of innovative practices being adopted by SNs to deliver routine school nursing services, there is a lack of evaluation or review of these practices (for young people and their families as well as SNs).	Research is needed to explore which innovative aspects of routine school nursing practice could (and should) endure post‐pandemic. Geographically focused research is needed due to the diversity of the SN role and healthcare provision globally. To undertake evaluations (from SN and service user perspectives) of practices which may be used more widely and extensively in post‐pandemic school nursing practice.
*The expanded school health offer*	There is evidence (anecdotal, informal and empirical) of SNs taking on various broader public health responsibilities during the pandemic, undertaken alongside their routine duties.	There is a paucity of empirical research exploring this topic. There is a lack of geographic representation across the literature.	While there is some evidence of innovative practices being adopted by SNs to deliver expanded services (i.e. broader public health) it is not clear what aspects (if any) may be maintained in routine school nursing services.	Research is needed to explore what aspects of SNs' expanded role will (and should) endure post‐pandemic.

Abbreviation: SN(s), school nurse(s).

#### 4.2.1 The continued school health offer

Themes that relate to the continued school health offer addressed how SNs adapted their practice to ensure they were able to continue providing formal and informal support to CYP and their families, and continue working closely with the MDT.

##### Engagement with CYP and families

Online or digital platforms were used for consultations or appointments with CYP (Barbee‐Lee et al., 2021; Gingell & Spencer, n.d.; Marrapese et al., 2021; Martinsson et al., 2021; Unknown, n.d.; Various, 2020b; Waters, 2021; White, 2020a, 2020b). Technology was also used to maintain contact with CYP (Booher, 2020), including the use of ChatHealth (Evans, 2020; Unknown, 2020c; Various, 2020a; Various, 2020b), online platforms (White, 2020b) and social media (Combe, 2020a). Telephone calls replaced in‐person health dialogues with CYP (Gingell & Spencer, n.d.; Martinsson et al., 2021; Various, 2020b), including for the conduct of routine health assessments and annual reviews (Tomkinson, n.d.; Unknown, 2020; Unknown, 2020c) and informal contacts with CYP (Combe, 2020a; Lee et al., 2021; Various, 2020a).

Technology and virtual platforms were also used in communications with parents (Barbee‐Lee et al., 2021; Evans, 2020; Flaherty, 2020; Marrapese et al., 2021; Martinsson et al., 2021; Waters, 2021; White, 2020a). The purpose of these virtual interactions included formal care plan reviews (Various, 2020b), digital interventions (Gingell & Spencer, n.d.) and provision of informal advice and support (Unknown, n.d.). SNs used telephone calls to communicate with parents for informal chats and check‐ins (Lee et al., 2021), consultations (Gingell & Spencer, n.d.; Martinsson et al., 2021; Unknown, 2020c) and annual reviews (Unknown, 2020d). Changes to routine screening consultations, such as sending questionnaires to parents, were reported (Barbee‐Lee et al., 2021). Email and post were also utilized with both CYP and parents (Tomkinson, n.d.; Unknown, 2020c; Unknown, 2020d).

Technology was reported as beneficial for ongoing CYP consultations (Martinsson et al., 2021), communications (Lee et al., 2021; Unknown, 2020c) and engagement (Gingell & Spencer, n.d.; Unknown, 2020). Remote options saved time for some SNs, CYP and families, although others felt the increased frequency of contacts increased demand (Martinsson et al., 2021). CYP were reported to prefer virtual over face‐to‐face contacts (Tomkinson, n.d.), citing ease of discussing personal/sensitive issues, and young carers appreciated the support this provided (White, 2020b). Informal feedback highlighted that SNs were able to gain a more in‐depth understanding of CYP's home situation and families' needs (Tomkinson, n.d.; Martinsson et al., 2021; White, 2020b). Though not ideal, conducting telephone assessments allowed CYP to share new concerns without delays to appointments (Unknown, 2020d).

However, virtual platforms could negatively impact the quality of SNs' conversations with CYP and families (Martinsson et al., 2021). Video conferencing capabilities were necessary because some SNs felt telephone contacts were not sufficient (Gingell & Spencer, n.d.). Also highlighted was the impact of digital poverty and resulting unequal access to services (White, 2021). Increased contact with students and families—often via virtual platforms—made additional demands on SNs' time (Martinsson et al., 2021; White, 2020b; Yip et al., 2020). Some reported a change in methods of communication to electronic only (Hoke et al., 2021).

SNs utilized alternative environments to ensure service delivery continued. Venues such as children's centers and empty offices were used (Barbee‐Lee et al., 2021; Evans, 2020). Various services were also delivered outdoors (Barbee‐Lee et al., 2021; Combe, 2020a; Fauteux, 2021; Martinsson et al., 2021; White, 2020a; White, 2021). Outdoor meetups (such as walk‐and‐talk sessions) were reportedly beneficial for a range of reasons including providing a ‘physical connection’ that was missing from virtual communication and being viewed positively by CYP and their families (Various, 2020b), resulting in improved attendance from CYP (Driscoll et al., 2021) and relaxed engagement (Evans, 2020). SNs also collaborated to distribute resources such as food and school supplies via drop‐offs and deliveries (Combe, 2020a; Fauteux, 2021; Robarge, 2021; Robinson, 2021). An extension to the timing of available services, such as routine vaccination access, was reported (White, 2021).

##### Education (CYP and families)

Virtual platforms were used to deliver routine health education (e.g. sexual health) to CYP and their families (Barbee‐Lee et al., 2021; Fauteux, 2021; Flaherty, 2020; Marrapese et al., 2021; Martinsson et al., 2021; Unknown, 2020b; White, 2020b).

##### Liaison with professionals

SNs used technology to facilitate engagement with professional networks, for example, virtually conducting Individual Education Planning, case conferences, core groups, child in need, disability support and safeguarding meetings (Flaherty, 2020; Unknown, n.d.; Unknown, 2020b; White, 2020b). Video conferencing was used for daily handovers, multi‐agency meetings (Gingell & Spencer, n.d.) and to connect with social work managers (Unknown, n.d.). Technology platforms were also used to help SNs carry out their roles and responsibilities with school‐based colleagues (Barbee‐Lee et al., 2021; Evans, 2020; Flaherty, 2020; Martinsson et al., 2021; Unknown, 2020b; Various, 2020b; White, 2020b; Yip et al., 2020). Digitally delivered training for school‐based colleagues around medical conditions was offered (White, 2020b).

Specific benefits of using technology for partnership work were reported as follows: the flexibility, time and travel cost savings, and greater attendance at meetings (Gingell & Spencer, n.d.). Virtual communication technology was also credited with providing new dimensions to relationship building and opened new communication channels, increasing the depth of discussions and improving the focus on children's needs (Unknown, n.d.). However, sometimes virtual platforms made it harder to discuss sensitive topics (Martinsson et al., 2021).

SNs offered emotional support to school staff (Lee et al., 2021), and similarly, peer meetings served as a way of providing mutual mental health support (Yip et al., 2020). SNs collaborated with their school‐based colleagues to address new challenges, such as working with adjustment counsellors to improve student ‘connectedness’ (Marrapese et al., 2021), with teaching staff to identify students at risk of chronic absenteeism, and with school counsellors to produce resource lists to meet families' needs (Combe, 2020a). SNs interacted with their peers in new ways such as convening support groups (Fauteux, 2021); in some cases technology was used to facilitate this interaction, including online support groups (Cogan, 2021), providing mutual support (Combe, 2020b), and using apps to communicate informally to help overcome professional isolation (Lee et al., 2021).

A virtual ‘town hall’ (where points of interest, policy and legislation are discussed) was also used by SN organizations to connect with members (Combe, 2020a), and an annual conference transitioned to a virtual environment (Robarge, 2021). The use of technology was beneficial to communication with other professionals (Lee et al., 2021; Martinsson et al., 2021). Increased technological use and advancement such as the increased use of online systems resulted in services being ‘safer, smoother and more efficient’ and access to online systems assisted SNs in protecting the most vulnerable (White, 2021, p. 50).

#### 4.2.2 The expanded school health offer

This theme captures the ways in which the SN role expanded beyond its pre‐pandemic remit, albeit in keeping with SNs' expertise as public health practitioners. This expanded role included a variety of novel and Covid‐19‐specific responsibilities.

##### Infection prevention and control: CYP and families

SNs took on numerous new Covid‐19‐specific roles and responsibilities with CYP. These included symptom monitoring and contact tracing (Combe, 2020b; Lee et al., 2021; Robinson, 2021; Sorg, 2020; Unknown, 2020a; Unknown, 2020b). Adaptations were made to existing daily practices, such as wearing personal protective equipment (PPE) for nebulizer administration (Barbee‐Lee et al., 2021); physical setups were altered to create isolation areas; and SNs were required to enforce public health isolation and quarantine requirements (Barbee‐Lee et al., 2021; Combe, 2020b; Ferrara, 2021; Lee et al., 2021; Robinson, 2021; Sorg, 2020). A telephone response service that parents (and schools) could contact for advice helped to relieve the pressure of calls to schools about Covid‐19 (Various, 2020b). Additionally, SNs sent reminder emails to parents encouraging them to screen their children (Combe, 2020b). SNs educated parents and carers on issues relating to Covid‐19 (Barbee‐Lee et al., 2021; Traut, 2020), and communication with parents often increased for pandemic‐related issues (Hoke et al., 2021; Lee et al., 2021; Unknown, 2020a; Yip et al., 2020).

Lesson plans to address Covid‐19‐related issues were developed (Booher, 2020). SNs produced pre‐recorded informational videos predominantly focused on Covid‐19 public health messaging (Combe, 2020a; White, 2020b; Yip et al., 2020), and sometimes shared these via social media platforms (Robarge, 2021; Various, 2020b). The videos had the reported benefit of providing advice and reassurance to CYP and parents (Various, 2020b). SNs were also involved in developing infographics to consolidate public health messages (Barbee‐Lee et al., 2021).

There were challenges to implementing new infection prevention and control measures, such as having to socially distance from younger children who may experience affectional neglect (Martinsson et al., 2021). Similarly, PPE requirements created barriers to expressing empathy and ‘building relationships with pupils’ (Evans, 2020, p. 7), while limited space in some schools made social distancing difficult (Lee et al., 2021; Sorg, 2020). SNs were also tasked with ensuring students' compliance with hygiene practices, which was reported to be challenging for example, among younger children and those with intellectual disabilities (Lee et al., 2021). Further, SNs found themselves ‘bearing the brunt’ of negative social responses to Covid‐19 regulations from parents (Cogan, 2021, p. 2) and teachers (Lee et al., 2021). The responsibility of ensuring families complied with guidelines, which carried implications for the wellbeing of the entire school community, was considerable (Robinson, 2021). SNs also faced the challenges of taking on new and extended roles with no additional training (Robinson, 2021; Yip et al., 2020), having to adapt practice in accordance with ever‐changing guidelines and a lack of information (Combe, 2020b; Lee et al., 2021), and balancing professional judgement with official guidance (Combe, 2020b) and school requests (Maughan et al., 2021). Some SNs also reported impacts on their ability to carry out their usual (non‐Covid‐related) duties (Combe, 2020b). Despite these challenges, SNs indicated that their role in preparing for students' return to school and developing/implementing Covid‐19 guidelines led to an increased professional visibility (Hansen, 2021; Lee et al., 2021; Maughan et al., 2021).

##### Infection prevention and control: Broader role

Beyond their daily work with CYP, SNs took on additional responsibilities to mitigate the risk of Covid‐19 transmission within the wider school community. These included planning for school reopening (Maughan et al., 2021; Robarge, 2021; Traut, 2020; Unknown, 2020b), providing staff with information and advice (Combe, 2020b; Lee et al., 2021; Traut, 2020; Various, 2020b), supporting management with data collection and usage (Hansen, 2021), supporting disinfection processes (Booher, 2020) and enacting quarantines or school closures (Combe, 2020b). SNs were ‘on‐call’ for conversations regarding evolving state guidelines (Traut, 2020). Lastly, SNs were involved in organizing new school‐located Covid‐19 vaccination events and required extensive peer and local authority collaboration (Park et al., 2021).

SNs assisted or collaborated with local and state‐level partners to pre‐empt and manage Covid‐19 outbreaks. These efforts focused on case monitoring and contact tracing (Marrapese et al., 2021; Robarge, 2021; Robinson, 2021; Traut, 2020), developing vaccination plans and follow‐ups (Kunz et al., 2022; Park et al., 2021), resource development and sharing (Kunz et al., 2022; Maughan et al., 2021; Unknown, 2020b), collaboratively investigating Covid‐19 outbreaks in the school community (Fauteux, 2021), acquiring PPE and planning for Covid‐related scenarios (Traut, 2020), and assisting in planning and guidance for students' return to school (Schwind, 2020; Traut, 2020). More broadly, SNs served as ‘a professional link’ between districts and local health centers (Booher, 2020, p. 22). SNs also networked and collaborated through ‘town halls’ (Maughan et al., 2021) and attended similar virtual events (Unknown, 2020a).

There were positive impacts of these collaborative efforts. A broad increase in cooperation within the school health service was reported (Martinsson et al., 2021). Increased interaction with building administrators led to a greater appreciation of SNs and their role (Hansen, 2021) and partnerships with local health boards elevated their profile (Flaherty, 2020). SNs garnered a ‘seat at the Executive Team table’ (Combe, 2020a, p. 186) and strengthened relationships with public health officials (Fauteux, 2021). There was variability in support and working practices (i.e working from home) across schools and managers (Martinsson et al., 2021). Similarly, professional conflicts emerged between some SNs and senior decision‐makers (Combe, 2020b; Sorg, 2020; Traut, 2020).

##### Innovation

Some SNs were directly involved in the development and implementation of new and innovative processes and tools, including IT systems such as password‐secured enrollment portals, electronic health records and e‐consent (Marrapese et al., 2021; White, 2021). For CYP, these included an electronic pass system to control the flow of students on campus (Bullard et al., 2021), a triage flow chart (Lee et al., 2021), and a standardized student wellness form (Barbee‐Lee et al., 2021). A state SN consultant collaborated to develop a system to identify and deliver PPE (Maughan et al., 2021). Online systems were also developed to meet specific new needs, including the tracking and reporting of staff and student symptoms and quarantine release procedures (Barbee‐Lee et al., 2021; Kunz et al., 2022; Traut, 2020), the provision of vaccine notifications and online text support services (White, 2021).

SNs also delivered regular ethical guidance to school‐based colleagues about the confidentiality of student information shared electronically (Marrapese et al., 2021). SNs were also involved in making adaptations to document sharing (Fauteux, 2021), data sharing and the use of hotlines for schools to report to the district (Hansen, 2021).

## 5 DISCUSSION

This scoping review identified 38 articles that described changes to the way SNs worked during the Covid‐19 pandemic. All articles were published in and about developed countries, predominantly the USA, followed by the UK, Hong Kong and Sweden. Analysis yielded two overarching themes: (1) *the continued school health offer*, which described the adaptations SNs made to continue providing their usual services during the pandemic, and (2) *the expanded school health offer*, which encapsulated additional SN roles and responsibilities resulting from Covid‐19.

The first themes details the ways SNs adapted their practice to continue working with CYP, families and the wider MDT when government restrictions limited on‐site service provision. There is a range of evidence supporting the effectiveness of digital health interventions with CYP, with efficiency and accessibility being notable advantages, though much of this evidence has focused on pharmacological and psychological interventions (see systematic and meta‐review by Hollis et al., 2017). Though born out of necessity, numerous articles described the benefits of shifting to virtual platforms for service delivery, with many citing improved communication with and/or access to CYP and families. This ongoing virtual communication also provided SNs with a new insight into CYPs' home environments, which may have facilitated a more holistic assessment of family dynamics and even the detection of abuse. Though our review did not identify articles discussing domestic abuse, the amplification of risk factors during the pandemic increased the vulnerability of many CYP to abuse and neglect at home (World Health Organization [WHO], 2020). SNs play a key role in detecting and responding to child abuse (Harding et al., 2019), and technology was vital for bridging the communication gaps created by the pandemic.

Despite many benefits, some articles highlighted the disadvantages of this increased reliance on technology, including digital poverty and the resulting exacerbation of existing health inequalities. A joint report from The United Nations Children's Fund (UNICEF) and International Telecommunication Union (ITU) (2020) showed that two thirds of school‐aged children globally have no internet connection at home. Evidence from a global systematic review indicates that other factors beyond connectivity can affect children's digital skills, including gender and socioeconomic status (O'Connor et al., 2016). These issues are important to take into account when considering the longevity of SNs' digital health offer. While our findings provide a snapshot of service providers' and users' experiences of using virtual platforms during the pandemic, it is important that these mechanisms for service delivery are thoroughly evaluated before decisions are made regarding their continued implementation (Perakslis & Ginsburg, 2021).

SNs' ongoing communication and working relationships with their school‐based colleagues and broader professional partners also changed during the pandemic. During a time where many CYP were increasingly vulnerable to deteriorating mental health (Panchal et al., 2021), exacerbation of existing physical health issues and problems at home (Cohodes et al., 2021), the importance of effective MDT collaboration was vital. Again, SNs' contact with other professionals largely relied on the use of virtual platforms. While much has been written on the effects and outcomes of using virtual platforms with service users, little has been published on the impact of this transition on health providers' interdisciplinary working. Given the likelihood that remote and virtual MDT collaboration will outlast the pandemic, this could be an important avenue for future research.

Beyond the impacts of the pandemic on SNs' traditional (i.e. pre‐pandemic) work, this review also identified various ways in which their role expanded. Though SNs around the world have long been key to shaping and delivering public health activities, the pandemic elevated this critical role. Many articles noted that SNs' central role in coordinating the pandemic response helped to strengthen working relationships, many of which arose as a direct result of Covid‐19, and provided SNs with new platforms to engage with policymakers. This improved policy‐level collaboration and influence is arguably one of the most important outcomes that needs to be sustained post‐pandemic. A recent guideline from WHO and the United Nations Educational, Scientific and Cultural Organization (WHO & UNESCO, 2021a) on school health services—the first of its kind—presents 87 specific interventions for improving the equity, quality and consistency of health services for school‐aged children globally. This guideline underscores the fundamental relationship between health and educational institutions, while also pointing out the inadequacy of SN coverage across many regions, despite policy calls for minimum staffing levels (WHO & UNESCO, 2021b).

Despite SNs' elevated profile, there is a wealth of evidence suggesting that SNs' rapidly evolving role had a detrimental impact on their ability to carry out routine practice. In a USA bulletin article, Robarge (2021) noted that mitigation efforts—something that would previously have occupied a small portion of SNs' time—suddenly became the predominant focus. Having responsibility for reviewing and implementing new school policies also meant that SNs bore the brunt of many peripheral social repercussions, such as the ‘infodemic’ (WHO, n.d.) and politicization of mask‐wearing and vaccinations (McIntosh et al., 2022). These challenges were compounded by the emotional burden of the pandemic, increased workloads and short staffing (variously exacerbated by SN redeployment). It is concerning to note that global SN numbers continue to fall short of what many authorities consider to be safe (WHO & UNESCO, 2021b).

Maughan and Luehr (2022) have drawn parallels between Covid‐19 and the 1918 Spanish Flu pandemic, noting that many of today's SN activities mirror practices from the early 20th century. Yet global development, new technology and nurses' ever‐increasing professional autonomy have meant that SNs were able to take on a leading public health role in this most recent global health crisis. Though this undoubtedly brought new challenges to an already stretched workforce, the profession was also strengthened by the increased visibility and reach of their expertise. SNs were quick to embrace new ways of working and proactively adapted their practice to meet new needs, including contributions to the development of numerous innovative solutions that will likely endure post‐pandemic. However, without adequate investment in staff and infrastructure, SNs' power to enact the changes envisioned by the WHO and UNESCO (2021b) and other global health authorities (NASN, n.d.; SAPHNA, 2021) will be limited.

### 5.1 Strengths and limitations

Though we had hoped to capture a range of international literature, the majority (*n* = 23) of our included articles came from the USA, limiting the generalizability of our findings. This will be especially true for low‐ and middle‐income countries where SN practice and coverage are more limited (or even non‐existent) (see WHO & UNESCO, 2021a). Our findings are further limited by the nature of the data we retrieved. The fact that the majority of our included articles were opinion and commentary pieces meant that most data were anecdotal, impeding our ability to undertake a systematic review. However, our findings reflect the global attention afforded to SN practice during the pandemic, highlighting a significant literature gap and uncovering important avenues for future research. The authors of this review are currently undertaking follow‐on research exploring SNs' experiences in more depth. By including grey literature we were able to capture a wider scope of evidence.

Our review was strengthened by its methodological rigor, including the comprehensive search strategy, screening and data extraction processes. Key expert stakeholders were actively involved throughout the process of developing, conducting and finalizing this review. Multiple researchers were involved in undertaking each of these processes, enhancing the reliability of our findings.

## 6 CONCLUSION

This scoping review presents global evidence describing how SNs' practices changed over the course of the Covid‐19 pandemic. The pandemic accelerated SNs' need and/or ability to devise creative solutions to emerging problems. SN knowledge and skills came to the fore, enabling continued delivery of child‐focused services alongside the additional demands of Covid‐19. Many of these innovative practices could be useful post‐pandemic. However, formal evaluation is needed to identify which practices may merit integration into routine practice. It is hoped that this review, together with other phases of this project and other research, will contribute to the discussion of innovative SN practices and the vital expert public health role of the SN.

## AUTHORS’ CONTRIBUTIONS

GC, JA, SB, TH, JT, DS: Made substantial contributions to conception and design, or acquisition of data, or analysis and interpretation of data; GC, JA, SB, TH, JT, DS: Involved in drafting the manuscript or revising it critically for important intellectual content; GC, JA, SB, TH, JT, DS: Given final approval of the version to be published. GC, JA, SB, TH, JT, DS: Agreed to be accountable for all aspects of the work in ensuring that questions related to the accuracy or integrity of any part of the work are appropriately investigated and resolved.

## FUNDING INFORMATION

This research was conducted as part of the project ‘Learning from the Covid‐19 pandemic in health and social care: implications for nursing practice’ kindly funded by the General Nursing Council for England and Wales (GNCT) 2021 grant call.

## CONFLICT OF INTEREST

The authors declare no conflict of interest.

### PEER REVIEW

The peer review history for this article is available at https://publons.com/publon/10.1111/jan.15504.

## Supporting information


Data S1

## Data Availability

The data that supports the findings of this study are available in the article and/or supplementary material of this article.
