# Supplementary material for: School nursing: New ways of working with children and young people during the Covid‐19 pandemic: A scoping review
Source: J Adv Nurs. 2022 Dec 21;79(2):471–501. doi: 10.1111/jan.15504 (PMC9877849; doi:10.1111/jan.15504)
Supplement: Supplementary file 1 — Data S1 [file JAN-79-471-s001.docx]

**Supplementary file 1 - Search strategy**

**Keyword search terms**

1. “School Nurs*” OR “school health nurs*” OR “community practitioner*” OR “specialist community public health nurs*” OR “public health nurs*” OR “school health”) in title/abstract

2. “Covid*” OR “pandemic” OR “corona*” OR "sars-cov-2”) anywhere in text

**MeSH terms**

1. School nursing
2. Covid-19 OR SARS-CoV-2

**PubMed**

1. TI/AB (“School Nurs*” OR “School health nurs*” OR “Community practitioner*” OR “Specialist community public health nurs*” OR “Public health nurs*” OR “School Health”) OR MH major topic (“School nursing”)
2. All fields (“covid*” OR “pandemic” OR “corona*” OR “corona*” OR "sars-cov-2”) OR MH major topic (“Covid-19” OR “SARS-CoV-2”)
3. Date range 2019-2021
4. Eng lang only

1 AND 2 AND 3 AND 4

**CINAHL via EBSCO**

1. TI/AB (“School Nurs*” OR “School health nurs*” OR “Community practitioner*” OR “Specialist community public health nurs*” OR “Public health nurs*” OR “School Health”) OR MH major topic (“School nursing”)
2. TX (“covid*” OR “pandemic” OR “corona*” OR “corona*” OR "sars-cov-2”) OR MH major topic (“Covid-19” OR “SARS-CoV-2”)
3. Date range 2019-2021
4. Eng lang only

1 AND 2 AND 3 AND 4

**British Nursing Database**

1. TI/AB (“School Nurs*” OR “School health nurs*” OR “Community practitioner*” OR “Specialist community public health nurs*” OR “Public health nurs*” OR “School Health”) OR mainsubject (“School nursing”)
2. TX (“covid*” OR “pandemic” OR “corona*” OR “corona*” OR "sars-cov-2”) OR mainsubject (“Covid-19” OR “SARS-CoV-2”)
3. Date range 2019-2021

No eng lang search

1 AND 2 AND 3

**Web of Science/Clarivate**

1. TI/AB (“School Nurs*” OR “School health nurs*” OR “Community practitioner*” OR “Specialist community public health nurs*” OR “Public health nurs*” OR “School Health”)
2. All fields (“covid*” OR “pandemic” OR “corona*” OR “corona*” OR "sars-cov-2”)
3. Date range 2019-2021

No eng lang search

1 AND 2 AND 3

**Proquest Health and Medicine**

1. TI/AB (“School Nurs*” OR “School health nurs*” OR “Community practitioner*” OR “Specialist community public health nurs*” OR “Public health nurs*” OR “School Health”) OR Mesh heading major topic (“School nursing”)
2. Anywhere (“covid*” OR “pandemic” OR “corona*” OR “corona*” OR "sars-cov-2”) OR mesh heading (“Covid-19” OR “SARS-CoV-2”)
3. Date range 2019-2021
4. Eng lang only

1 AND 2 AND 3 AND 4

**Nursing & Allied Health (Proquest)**

1. TI/AB (“School Nurs*” OR “School health nurs*” OR “Community practitioner*” OR “Specialist community public health nurs*” OR “Public health nurs*” OR “School Health”) OR Mesh subject (“School nursing”)
2. Anywhere (“covid*” OR “pandemic” OR “corona*” OR “corona*” OR "sars-cov-2”) OR mesh subject (“Covid-19” OR “SARS-CoV-2”)
3. Date range 2019-2021
4. Eng lang only

1 AND 2 AND 3 AND 4

**Proquest Public Health**

1. TI/AB (“School Nurs*” OR “School health nurs*” OR “Community practitioner*” OR “Specialist community public health nurs*” OR “Public health nurs*” OR “School Health”) OR Mesh subject (“School nursing”)
2. Anywhere (“covid*” OR “pandemic” OR “corona*” OR “corona*” OR "sars-cov-2”) OR mesh subject (“Covid-19” OR “SARS-CoV-2”)
3. Date range 2019-2021
4. Eng lang only

1 AND 2 AND 3 AND 4

**Proquest Family Health**

1. TI/AB (“School Nurs*” OR “School health nurs*” OR “Community practitioner*” OR “Specialist community public health nurs*” OR “Public health nurs*” OR “School Health”) OR mainsubject (“School nursing”)
2. Anywhere (“covid*” OR “pandemic” OR “corona*” OR “corona*” OR "sars-cov-2”) OR mainsubject (“Covid-19” OR “SARS-CoV-2”)
3. Date range 2019-2021
4. Eng lang only

1 AND 2 AND 3 AND 4

**PsycInfo**

1. TI/AB (“School Nurs*” OR “School health nurs*” OR “Community practitioner*” OR “Specialist community public health nurs*” OR “Public health nurs*” OR “School Health”) OR MH major topic (“School nursing”)
2. All text (“covid*” OR “pandemic” OR “corona*” OR “corona*” OR "sars-cov-2”) OR MH major topic (“Covid-19” OR “SARS-CoV-2”)
3. Date range 2019-2021
4. Eng lang only

1 AND 2 AND 3 AND 4

**Cochrane Database for SRs**

1. TI/AB (“School Nurs*” OR “School health nurs*” OR “Community practitioner*” OR “Specialist community public health nurs*” OR “Public health nurs*” OR “School Health”)
2. All text (“covid*” OR “pandemic” OR “corona*” OR “corona*” OR "sars-cov-2”)

1 AND 2
